# 1441. Significant Decrease in the Incidence Rate of Common Outpatient Upper Respiratory Tract Infection Diagnoses per Clinic Visit in the First Respiratory Season of October 2020 to March 2021 During the Covid-19 Pandemic. A Report From an Outpatient Antimicrobial Stewardship Program at a community hospital in Brooklyn

**DOI:** 10.1093/ofid/ofab466.1633

**Published:** 2021-12-04

**Authors:** Jilan M Shah, Olga Badem, Zeyar Thet, Thinzar Wai, Karthik Seetharam, Olawale Akande, Sherin Pathickal, Maxine Orris, Ngozi Kanu, Laurie Ward, Tanveer Mir

**Affiliations:** 1 Wyckoff Heights Medical Center, Brooklyn, New York; 2 Wyckoff Heigh Medical Center, Brooklyn, NY

## Abstract

**Background:**

As part of our outpatient Antimicrobial Stewardship Program, we do surveillance of diagnoses and antibiotic use for common upper respiratory tract infections such as acute upper respiratory tract infection, acute bronchitis, sinusitis, and pharyngitis. We sought to evaluate the impact of the Covid-19 pandemic on the incidence rate of upper respiratory tract infection diagnoses per clinic visit during October 2020 to March 2021 season compared to the three prior respiratory seasons. We also sought to reflect of impact of increase in televisits and overlapping symptoms of COVID 19 and upper respiratory tract infections.

**Methods:**

Our cohort study extending from October 2017 to March 2021. We collected number of diagnoses of upper respiratory infections and number of unique clinic visits during four consecutive respiratory seasons at our primary care sites via electronic health records.

**Results:**

During the recent October 2020 to March 2021 respiratory season which coincided with the second NYC Covid-19 wave, we had 11569 unique clinic visits and 39 diagnoses of an upper respiratory tract infection - incident rate of 1.29. In the three prior respiratory seasons combined, we had 40939 unique clinic visits and 833 diagnoses of an upper respiratory tract infection – incident rate of 1.49. The incident rates showed a dramatic decline using the test based method and the chi square-statistic p< 0.0001 with an incident rate ratio using a poisson exact method of 6.0359. Statistical comparisons of the current season to each prior individual season yielded similar results. The percentage of Tele-visits during the current season was 19% compared to 0% in the 3 prior seasons.

**Conclusion:**

During the first respiratory season from October 2020 to March 2021 in midst of the Covid-19 pandemic which also coincided with the second Covid-19 wave in New York, we saw a statistically significant decrease in incidence of common upper respiratory tract infection diagnoses per clinic visit compared to the three prior respiratory seasons. Overlapping signs and symptoms of upper respiratory tract infections and Covid-19 with the added percentage in Tele-visits did not cause an increase in incidence rates of upper respiratory tract infection diagnoses. Covid-19 related mitigation efforts may have played a role.

**Disclosures:**

**All Authors**: No reported disclosures

